# Individual variation in play in young chickens – assessment and connection to affective state and personality

**DOI:** 10.1038/s41598-025-34437-x

**Published:** 2026-01-06

**Authors:** Rebecca Oscarsson, Louise Hedlund, Austeja Rutkauskaite, Per Jensen

**Affiliations:** Linköping University, AVIAN Behaviour Genomics and Physiology Group, Linköping University, IFM Biology, 581 83 Linköping, Sweden

**Keywords:** Play behaviour, Individual variation, Affective state, Personality, Ecology, Ecology, Neuroscience, Psychology, Psychology, Zoology

## Abstract

The extent of individual variation in play frequency, and its association with affective state and personality is unexplored. We evaluated two different methods to study individual variation: video stimulation and individual consistency across different group constellations. In both cases, the birds were moved from the home pen to identical play arenas, and the play sessions were video recorded. Furthermore, a cognitive judgement bias test was performed, and in one group, personality tests were also performed. Large variation in individual play frequency was observed with both methods. Video stimulation did elicit some play behaviour, but the play frequency was lower than expected, and no social play was observed. Individual play frequency was relatively consistent across test days when chicks were allowed to play in different groups, suggesting a possible genetic influence on play motivation. No connection between individual play frequency and affective state was found, suggesting that play is weakly associated with fundamental state of affect in chicks. Furthermore, the results revealed a negative correlation between food exploration and total play in females, and between food exploration and affective state in males. This suggests that food motivation was greater in females who played less and in males exhibiting greater optimism.

**Supplementary Information:**

The online version contains supplementary material available at 10.1038/s41598-025-34437-x.

## Introduction

Play occurs in many animal taxa, including birds^1^. In the absence of an established definition, five criteria are commonly used to assess play^2^, stating that; first, the behaviour appears to lack immediate function in the occurring context; second, it is rewarding and/or pleasurable to the performer; third, it diverges in structure or temporal organization from functional expression of behaviour; fourth, it is performed repeatedly, however in a non-stereotypic nature, throughout parts of the ontogeny or more; fifth, it occurs mainly when the animal is in good health and free from stress. For non-human animals, three main categories of play are often adapted; locomotor play, social play, and object play^3^, and with the right conditions, young chickens will engage in behaviours of all three categories^4–6^. The literature on play in chickens is growing, but the extent of individual variation has not been addressed in detail.

Behavioural differences among individuals have been studied extensively in non-human animals over the last decades, and there is now much agreement that phenotypic variation is prevalent both at the inter- and intra-individual level^7^. Although minor differences in definitions occur, several terms are often used interchangeably when describing consistent behavioural differences between individuals, e.g., temperament, animal personality, coping style, and behavioural syndrome^8,9^. Animal personality has been described as individual differences in behaviour consistent across time and/or contexts^10^. Some major personality trait categories put forward include activity, exploration-avoidance, shyness-boldness, aggressiveness, and sociability^9^. It is known that chickens have different personalities, for instance, consistent behavioural differences have been identified for boldness and exploration^11,12^. Playfulness has been claimed to be a personality trait in dogs^13,14^, and individual playfulness has been found to be somewhat consistent in rats^15^. However, research on consistency in individual play frequency is limited across species, including chickens, and remains to be thoroughly investigated.

Knowledge about individual variation in motivation to play is important for animal welfare. Play behaviour shows potential of being used as an indicator of positive welfare, as animals supposedly engage in playful activity mainly when in a positive affective state^16^. In pigs, locomotor play is associated with positive affective state^17^, and in spotted pacas, object play can potentially be used as an indicator of positive emotional state^18^. Although positive affect seems to increase play in some animals^19^, exceptions occur, and the relationship is not straightforward^20^. Early weaning has been found to increase object play in domestic kittens^21^, and hatchery stress has been found to increase the overall play frequency in laying hen chicks^4^. In chickens, play has been used as a measure of positive welfare^22,23^, although the relationship between motivation to play and affective state has not been investigated in this species.

The affective state of an animal can be assessed, e.g., with a cognitive judgement bias test, which measures if the animal is in a so called optimistic or pessimistic state^24^. To study this, animals are often trained to associate two different stimuli with either a positive (e.g. a food reward in a white bucket) or a negative (e.g. an empty black bucket) outcome. Then, the animal is presented with an ambiguous stimulus (in this case a grey bucket), and whether it interprets this rather as the positive or the negative stimulus serves as a measurement of its affective state. To avoid training, the use of ecologically relevant stimuli has been explored, and Salmeto, et al.^25^ were first to try this in chicks. Our research group adopted this method, relying on the innate perception of two-dimensional pictures of a chick, an aversive owl, and a morph of the two, and has used it successfully in several previous studies^26–28^.

Personality and play, and particularly the relationship between the two, are understudied areas of research. The play-personality relationship was explored in two recent studied on ground squirrels where social play was found to predict both docility and caution^29,30^. In chickens, nothing is yet known about this relationship. In Red Junglefowl (RJF), the ancestor of all domesticated chickens, various cognitive aspects have been found to be associated with personality^31,32^, and White Leghorn (WL) chicks that had played more socially were bolder and more explorative^33^. We previously found play ontogeny in young chickens to be affected by both domestication^4^ and tameness^6^. The results varied for the different play categories, but overall, domesticated WL played more than ancestral RJF, and RJF selected for low fear of humans played more than those selected for high fear of humans. These differences open the possibility to map the genetic architecture of individual variation in playfulness, using an advanced intercross between WL and RJF. In such an intercross, segregation of phenotypical traits is likely to occur, and large individual variation is expected. However, genetic mapping requires that play can be measured at the individual level. This is associated with some difficulties: Firstly, when left without conspecifics, chicks experience a great deal of stress and will not play, and secondly, when in groups, individuals are bound to influence each other, as many of their play behaviours involve social elements. Therefore, we aimed to evaluate the use of two different methods as potential ways of studying individual variation in play: (1) video stimulation, and (2) individual consistency across different groups of conspecifics. In addition, we explored the link between individual play motivation, affective state and personality. The work is presented in three parts: (1) Effects of video stimulation, (2) Individual consistency in play, and (3) The relationship between play, affective state and personality.

### Part 1: effects of video stimulation

In Part 1, we present the evaluation of whether young chickens can be stimulated to play using video stimulation that would simulate social contact. Before the start of the experiment, we created a play stimulation video, portraying a group of chicks engaging in regular play bouts. The play arenas were set up with one screen each and before the test sessions, the birds were habituated to spending time alone in the arenas with the video. Then, for two consecutive days, the birds were tested first alone with the video and then in pairs without any video.

## Methods

### Ethical statement

The study received approval from the Linköping Council for Ethical Licensing of Animal Experiments (license no. 10492–2023) and was conducted in compliance with the ARRIVE guidelines and local regulations.

### Animals and housing

The birds (n = 32, 15 males and 17 females) were the F22 progeny of a RJF × WL advanced intercross. The F0 animals were one male RJF and three female WL. The male originated from a zoo population and the female line (SLU13) from a Scandinavian laying hen crossbreeding project. Detailed information about the origin of the birds is available in^34^. Ownership of this line is held by Linköping university. Birds of this intercross has a large variation in plumage colour, ranging from white to black. When necessary, lighter individuals were colour marked to enable individual identification from above.

The experiment was carried out in research facilities of Linköping university. The eggs were incubated and hatched in darkness, in one and the same incubator set to 37.8 °C, 55% relative humidity, and with hourly rotation. Three days before hatch, the eggs were moved to hatching trays with hatcher settings 37.5 °C and 65% relative humidity.

The chicks were housed in mixed-sex groups of eight individuals. The home pens were solid floor cages (W × L × H: 0.7 × 0.68 × 0.57 m) provided with saw dust, a heat roof, and ad lib access to food and water. At 30 days of age, the heat roofs were removed and replaced with perches. The cages were situated in the same room as the test arenas, less than 5 m away.

When the experiment had ended, the females were adopted out, and the males were sacrificed through rapid decapitation.

### Experimental setup

The testing was conducted in completely enclosed play arenas, considerably larger than the home pens (L × W × H: 1.17 × 0.8 × 1 m). Three adjacent arenas were set up with a computer screen in one short end, allowing three individuals to be tested simultaneously. The floor of each arena was covered with sawdust. The home cages and test arenas were all in the same lab room. For all test procedures, the chicks were carefully caught and placed in cardboard boxes, thereafter, moved over to the test arenas and gently put in the middle of them, with the lights turned off. Pictures of the test arena setup are shown in Figure S1-2, Supplementary Information 1.

### Play stimulation video

The footage used for the stimulation videos was filmed prior to the start of the experimental period, in the same arenas. For this, a group of two RJF and two WL were used. From the video footage, two videos were edited: one control video and one play video. In the control video, the chicks were seen engaging in other activities than play, such as scratching and pecking in the sawdust, and preening. In the play video, one play session lasting between 5 and 25 s occurred every minute, i.e. 15 play sessions in total. Behaviours from all three play categories were included, and each play session entailed behaviours from one or multiple categories. The stimulation was only visual as the video had no sound. The play video is provided in Supplementary Information 4.

### Habituation

The goal of the habituation phase was for the chicks to be comfortable being alone in the arenas with the play stimulation video at the time of testing, i.e., day 28. Habituation was conducted the following days; day 5 and 6 in groups of 8 (full cage), day 6–8 in groups of 4 (half a cage), day 12–16, 18 and 21 in pairs, and day 22–27 individually. The groups and pairs were always the same individuals. During habituation, the control video was shown day 13–16, and the video with play was shown from day 18 and onwards. To stimulate object play, a fake worm (from day 22, measuring 3 × 165 mm) and four live mealworms (from day 26) was placed in the middle of each arena prior to the start of each session. Each session lasted 15 min.

### Play tests

All chicks were tested alone with the play video and in pairs without video. The video stimulation play test was conducted on day 28 and the test had the same setup as the habituation session on day 27, i.e. one chick at a time was video recorded for 15 min with the stimulation video playing throughout the whole test. The arenas were equipped with a fake worm and four live mealworms in a small transparent bowl. As the test started, the light was turned on, and simultaneously, the video started playing. For comparison, the chicks were tested in pairs on day 29, with the same setup, except without any video. The same pairs as during habituation were used. The full play ethogram is provided in Supplementary Information 3.

### Sampling and data analysis

The chicks were video recorded for 15 min, both during video stimulation and in pairs. The behaviours were then recorded in time segments of 5 s, using 1/0 sampling. For each chick and every segment of 5 s, it was recorded whether they engaged in each of the behaviours in the ethogram or not. For every individual and test, each behaviour could therefore get a score between 0 and 180. Each individual therefore got three play scores: one for the video condition, one for the pair condition and one mean play score, i.e. the mean play frequency of the video and pair condition combined. No software was used for the scoring of play behaviours. Shotcut was used as video player, and the same observer (the first author) scored all videos.

We calculated Pearson correlation coefficients to assess the connection between the occurrence of total play in the different conditions. The graphs were made in R version 4.3.0 using the ggplot2 package, and the statistical analyses were computed in SPSS 29.0.0.0.

## Results

**Fig. 1:**
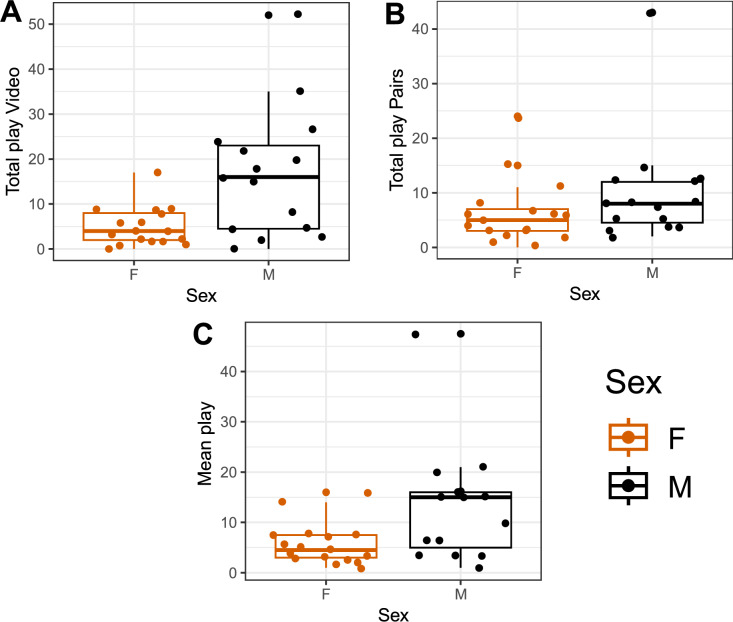
Total number of play observations per individual per 15 min in conditions (**A**) video stimulation, (**B**) pairs, and (**C**) mean play frequency of both conditions. The graphs are box- and dot-plots displaying individual values (dots), medians (horizontal line), lower and upper quartile range (box). Whiskers represent the range of values within 1.5 × the interquartile range (IQR) from the lower and upper quartiles. Data points beyond this range are plotted individually as outliers.

**Fig. 2:**
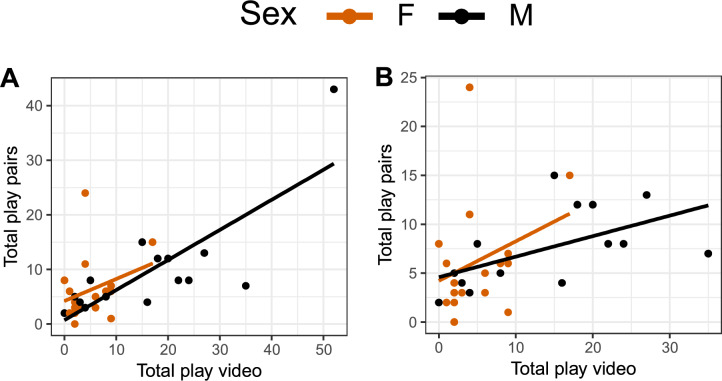
Correlation between total number of play observations in the video and pair condition per 15 min of (**A**) all individuals, (**B**) without one high-playing male. Each dot represents one individual.

Individual variation was observed in both the video and pair condition (Fig. 1A,B). For males, there was a significant positive correlation between play with video and play in pairs (Fig. 2A; males: r = 0.79, p < 0.001; females: r = 0.30, p = 0.25). The correlation remained significant when one high-playing male was removed (Fig. 2B; males: r = 0.56, p = 0.04). In males, this correlation stems from a significant positive correlation between the video and pair condition for both locomotor and object play, correlations that were not present in females (Figure S3, Supplementary Information 1).

## Discussion

In Part 1, we aimed to explore whether video stimulation can be used to stimulate play in young chickens. Individual variation in play was observed in both the video and pair condition. The males played more than females, in line with our previous findings^5^. The play consisted of mainly locomotor and object play, and video stimulation appeared more successful as a measure of individual variation in play in males than females. As could be expected, no social play occurred in the video condition, since the chicks were alone in the arena. This is a clear, potentially inevitable, disadvantage with this method. Chickens are known to perceive and respond appropriately to video images in different contexts^35,36^, but to our knowledge, this is the first time the use of video stimulation to stimulate play in chicks has been explored. When successfully applied, video stimulation has great benefits as it offers a controlled and standardised manipulation of an environment. Despite our effort, we believe that the chicks were not completely comfortable being alone in the arenas, hence affecting their motivation to play. Other behaviours than play were not scored, but the birds were perceived as less active in general. Based on our previous experience from observing play in groups of young chickens^4–6^, the frequency was overall lower in both conditions, than what is expected to observe in a group setting. Modifications to the setup, such as even more habituation to the test arena and longer exposure time to the video, could potentially increase the play motivation. However, such modifications are time consuming and were not feasible in this experiment.

In conclusion, video stimulation showed some potential as a way of stimulating play in young chickens, but not enough to motivate the use of this method for large-scale individual phenotyping.

### Part 2: Individual consistency in play

In Part 2, we present the investigation of individual consistency in play, addressed by allowing chicks to play in different group constellations. The same play arenas as in the video stimulation experiment were used (but without the video screen). After a period of habituation, for three consecutive days, the birds were tested in groups of three, with different familiar conspecifics each time. The procedure was repeated two weeks in a row around the play peak period.

## Methods

### Animals and housing

The birds (n = 45, 20 males and 25 females) were of the same RJF × WL intercross and came from the same parental flock as those used in part 1.

Incubation and housing occurred under the exact same conditions as in part 1, although in this experiment, to allow the creation of three groups of three individuals within each cage, the birds were housed in mixed-sex groups of nine individuals each. The cages were situated in the same room as the test arenas, less than 5 m away.

When the experiment had ended, all birds were moved to another research facility of Linköping university.

### Experimental setup

The play tests were conducted in the same arenas as in the video stimulation experiment, but this time, no screens were set up. The floor was covered with sawdust. For all habituation and test procedures, the chicks were carefully caught and moved to the test arenas in cardboard boxes, then gently put inside them, with the lights turned off.

### Habituation

As in the video stimulation experiment, the goal was for the chicks to be comfortable in the arenas at the time of testing, i.e., day 28. Habituation was conducted twice per week starting at 7 days of age. More specifically, habituation occurred on day 7 (in groups of nine, full cage) and on day 9, 13, 16, 20, 23 and 27 in random groups of three (1/3 cage). From day 16 and onwards, for each habituation session, the groups were provided with a fake rubber worm (3 × 165 mm) and four live mealworms in a small transparent bowl.

### Play tests

The play tests occurred at around four and five weeks of age, to cover what we previously found to be the peak play period^4–6^. The chicks were tested once per day three days in a row (day 28–30 and day 35–37) in groups of three, each time with different conspecifics. The different group constellations were created within the home cages; hence the chicks were always familiar with each other. Since we could not sex the chicks at hatch and therefore were unable to control the male–female ratio in the cages, the play groups were of both mixed and same sex. All birds from one cage, i.e. three groups, were tested at the same time in parallel arenas. To stimulate object play, each group was provided with one fake worm and four mealworms (the same objects as during habituation, and the same as were used in part 1). The fake worm and the bowl with mealworms were placed in the opposite end of the arenas as to where the chicks were introduced. The light in the arenas was turned on as the test session started. The aim was to identify how stable the individual play frequency was and evaluate how well this method captures this.

### Sampling and data analysis

For all test sessions, the chicks were video recorded for 15 min and the behaviours were scored in the same way as for the video stimulation experiment, i.e. in time segments of 5 s, using 1/0 sampling. Hence, each individual got one play frequency score per play category for each of the six test days. No software was used for the scoring of play behaviours. Shotcut was used as video player, and the same observer (the first author) scored all videos.

To evaluate how consistent the individual play frequency was, we calculated Pearson correlation coefficients to assess the connection between the occurrence of total play for each of the three days, for both weeks of play tests. The results for the different play categories are provided in Figure S4-6, Supplementary Information 1. Additionally, the total mean play score for each individual and week was calculated, and the correlation between the two weeks was assessed using Pearson’s correlation analysis. The graphs were made in R using the ggplot2 package, and the statistical analyses were performed in SPSS.

## Results

**Fig. 3:**
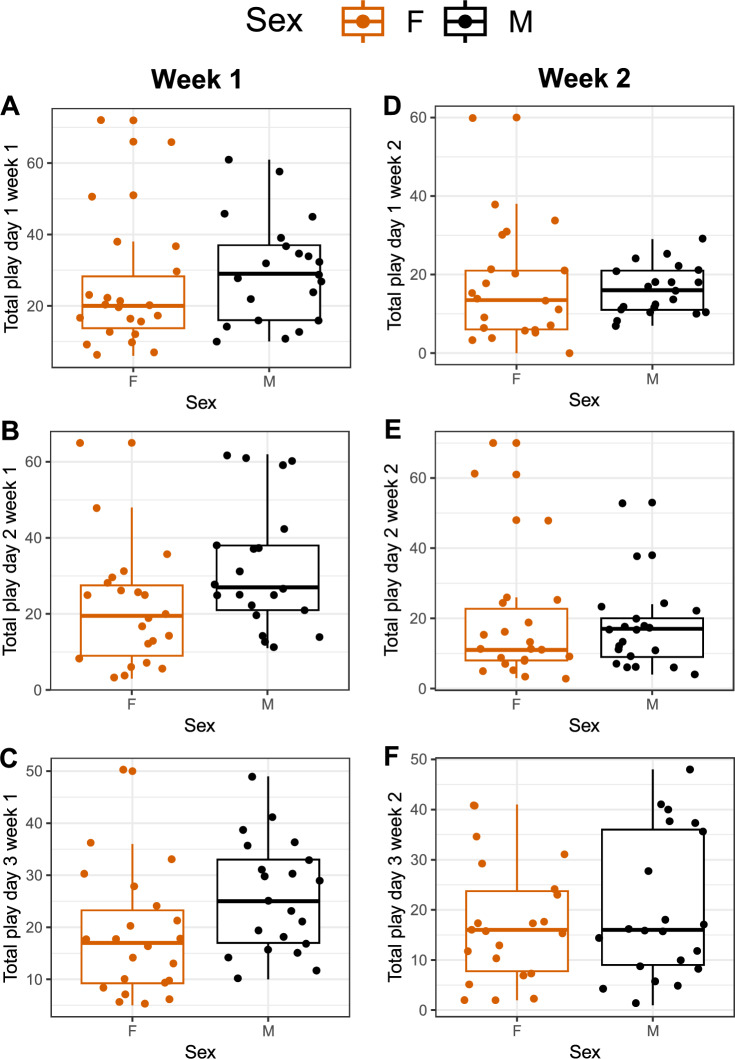
Total number of play observations per individual per 15 min of (**A**) day 1, (**B**) day 2, and (**C**) day 3, the first week of testing, and (**D**) day 1, (**E**) day 2, and (**F**) day 3, the second week of testing. The graphs are box- and dot-plots displaying individual values (dots), medians (horizontal line), lower and upper quartile range (box). Whiskers represent the range of values within 1.5 × the interquartile range (IQR) from the lower and upper quartiles. Data points beyond this range are plotted individually as outliers.

**Fig. 4:**
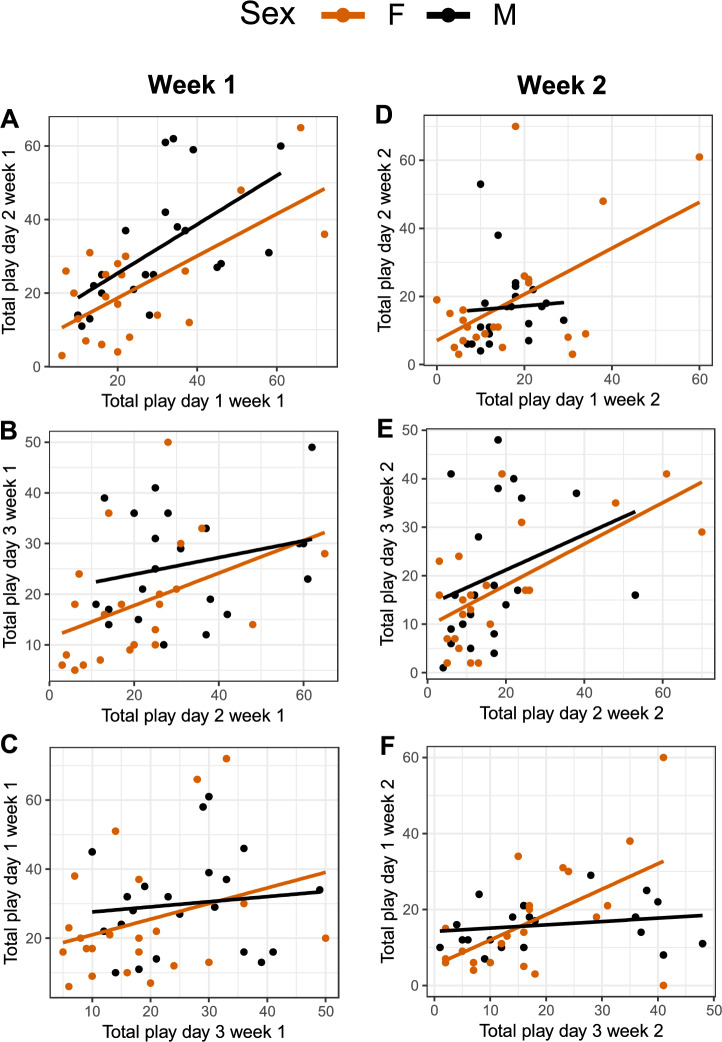
Correlation between total number of play observations per individual per 15 min of (**A**) day 1 and 2 week 1, (**B**) day 2 and 3 week 1, (**C**) day 3 and 1 week 1, (**D**) day I and 2 week 2, (**E**) day 2 and 3 week 2, (**F**) day 3 and 1 week 2. Each dot represents one individual.

Large individual variation was observed for all three days of play tests, during both weeks (Fig. 3A-F). For the first week of play tests, there was a significant positive correlation between play scores on day 1 and day 2 for both males and females (Fig. 4A; males: r = 0.59, p = 0.005; females: r = 0.68, p < 0.001). Between day 2 and day 3, the correlation was only significant for females (Fig. 4B; males: r = 0.26, p = 0.26; females: r = 0.45, p = 0.03), and no correlation was present between day 3 and day 1 for either of the sexes (Fig. 4C; males: r = 0.11, p = 0.64; females: r = 0.32, p = 0.14).

For the second week of play tests, there was a significant positive correlation between play scores on day 1 and day 2 for females (Fig. 4D; males: r = 0.06, p = 0.80; females: r = 0.56, p = 0.006), and it remained significant for females between day 2 and day 3 (Fig. 4E; males: r = 0.29, p = 0.20; females: r = 0.60, p = 0.002), and between day 3 and day 1 (Fig. 4F; males: r = 0.21, p = 0.37; females: r = 0.46, p = 0.03).

**Fig. 5:**
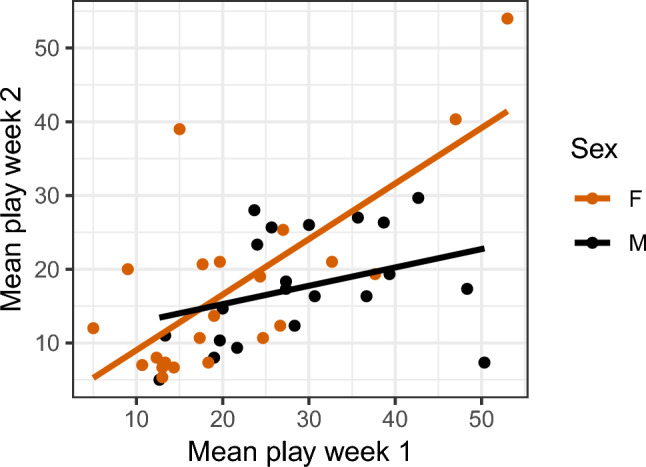
Correlation between the mean individual play frequency of the three days of play tests the first and second week of testing. Each dot represents one individual.

There was a significant positive correlation between the mean play frequency (mean of the three test days) in the first and second week of play tests for females (Fig. 5; r = 0.73, p < 0.001), but not for males (Fig. 5; r = 0.35, p = 0.12).

Correlations for the different play categories are provided in Figure S4-6, Supplementary Informationl 1. Object play was consistent across days for female, but not males. Locomotor play showed consistency for both sexes, while the social play frequency was not consistent for either sex.

## Discussion

In Part 2, we aimed to explore individual consistency in play in young chickens. The individual play frequency was somewhat consistent, for females in particular, but was probably affected by factors such as age and group constellation. Overall, the play frequency was slightly lower the second week of testing, most likely reflecting an age effect. Our previous work revealed that the total play frequency peaks around 30–40 days of age, earlier in WL than in RJF^4^. Hence, the play peak was expected to occur around this age, although, in the RJF × WL intercross that was used in the present study, a large individual variation in overall play frequency, as well as time point for when the play frequency peaks, was expected.

We have previously showed that male chicks play more than female in same sex groups^5^. In the present study, the sex ratio in the groups varied between the days (since each day had a new group constellation), and this is likely to have influenced the individual play frequency. For instance, if a male was in a group with two females, this may have affected the amount of social play performed by that individual. With the setup used, sex ratio is difficult to control for since we are unable to sex the intercross chicks right after hatch.

To date, little is known about how consistent individual play levels are across different ages and contexts in animals. Consistent individual differences in playfulness have been identified in rats across age and motivational state^15^. However, only male rats were used, and the contexts were play in pairs with and without prior isolation, tickling by a human experimenter, and spontaneous play in the home cage. Although consistency was found, the different play types and contexts were unrelated, hence, the authors believe that different play types represent motivationally distinct systems. A similar experimental setup could tentatively be used in the future to gain deeper understanding of individual motivation to play in chickens. Moreover, in rats, consistent individual differences across days have been found for social play in both males and females, when paired up with an unfamiliar individual of the same sex^37^. The rats appeared to consistently adjust their play behaviour in response to the behaviour of the partner, hence displayed a somewhat stabile play frequency overall. In contrast, we did not find social play in chickens to be consistent across test days. This could potentially be explained by the fact that we studied familiar birds in groups of three instead of pairs, and that the sex ratio in our groups varied.

In conclusion, the consistency of the individual play frequency varied for different play categories. Overall, the variation was somewhat consistent across test days, more so for females than males. This suggests that a genetic basis for play motivation could be present, at least for some play behaviours. The method therefore shows promising results with respect to the possibility to quantify individual variation in play.

### Part 3: The relationship between play, affective state and personality

In part 3, we present our findings regarding the relationship between play, affective state and personality. At the end of the two play experiments presented in part 1 and 2, a cognitive judgement bias test (CJB) was performed on all chicks. Additionally, at the end of the experiment investigating individual consistency in play, all chicks were exposed to three different personality tests meant to explore the traits sociability and exploration. The tests were a social reinstatement test, an exploration test, and a social versus foraging test.

## Methods

### Cognitive judgement bias test

**Fig. 6:**
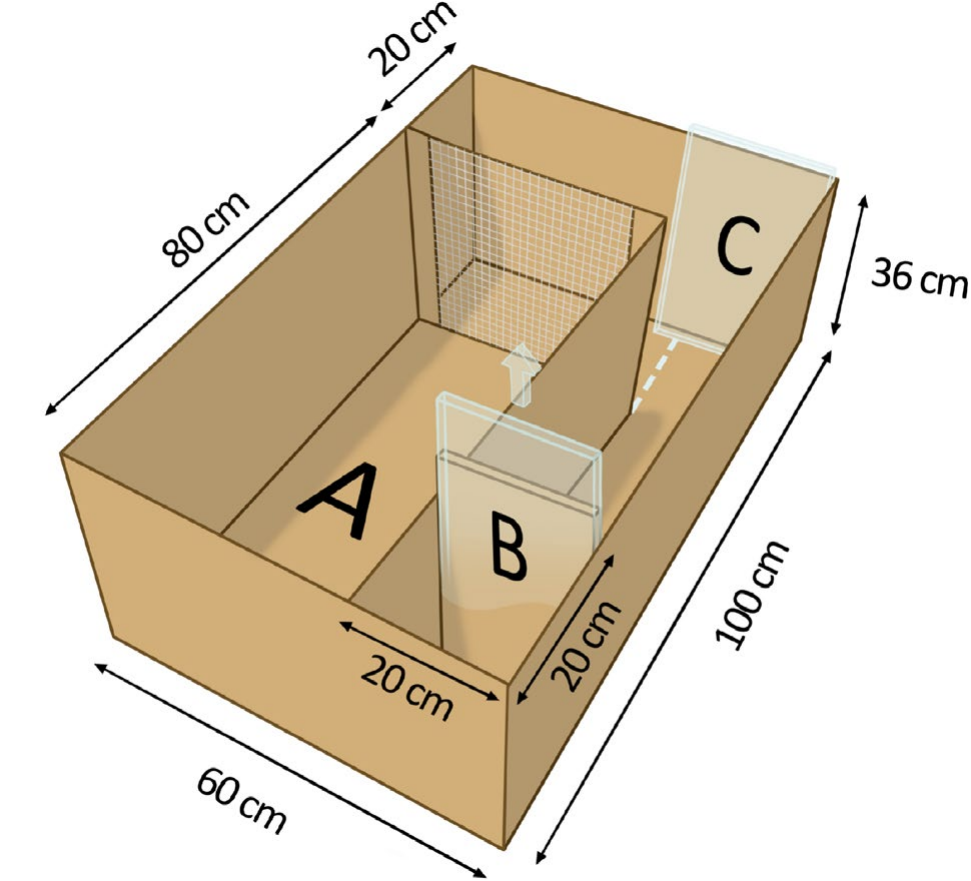
Schematic drawing of the cognitive judgement bias arena. The letters mark the; **(A)** conspecific area, **(B)** start box divider, and **(C)** stimulus position. The arena was made out of cardboard and the bottom was covered with sawdust during testing.

All chicks were exposed to a CJB test on day 33 (video stimulation) and day 38 or 39 (individual consistency in play). We adopted the method developed by Salmeto, et al.^25^ and further adapted by Hedlund, et al.^26^. The test arena set-up is illustrated in Fig. 6. The arenas were made of cardboard and four or five birds at a time were moved to the conspecific area. One at a time, the chicks were placed in the start box, and after a duration of 10 s, released to explore and move through the alley. To regain contact with the conspecifics, the chicks had to cross an imaginary goal line and pass a stimulus at the end of the alley, that could be perceived as positive (a mirror), near positive (a chick), negative (an owl) or ambiguous (a morph between the chick and the owl). The stimulus cues are shown in Figure S8, Supplementary Information 1. Each chick was tested on all four stimuli, and since we were interested in individual differences, these were presented in the same order for all individuals. In the video stimulation experiment, the order was mirror-chick-owl-morph, and in the experiment investigating individual consistency in play, the order was mirror-owl-chick-morph. The change was decided because the chicks in the video stimulation experiment had an unexpected long latency to the chick stimulus. All four or five conspecifics were exposed to each stimulus before moving on to the next. The maximum test time for each stimulus was 5 min, and if a chick had not reached the goal line within this time a latency of 5 min was recorded. The morph was created in Morpheus Photo Morpher v.3.17 Standard. All images were printed on a white background and were of the same size; approximately the same as the chicks being tested.

### Social reinstatement

The social reinstatement test was conducted on day 41 in six test arenas used in parallel. The arenas measured 1.17 × 0.4 m. One short end was parted off by wire mesh to create a conspecific area. Three birds at a time, all from the same cage, were placed in each conspecific area and left there to habituate for 10 min. Thereafter, one individual at a time was taken out and inserted through a small lid in the opposite end, while the lights were turned off. The light was then turned on and each bird was allowed to explore the arena for 10 min. The trials were video recorded using overhead cameras. From the videos, latency to leave start zone, latency to enter social zone, latency to first zone crossing, number of zone crossings, and time spent in social zone, was scored. A picture of the test set up (Fig. S10), as well as an ethogram with scoring criteria, is available in Supplementary Information 1.

### Exploration test

The exploration test was conducted on day 42, in the same arena as the play tests. The floor was covered with sawdust. Four round black dishes with sand were spread out evenly along each side of the arena, and two of the dishes were bated with 5 g of mealworms. One unique novel object was placed in each corner. The birds were placed individually in the centre of the arena with the lights turned off. The lights were turned on as the test started and each bird was allowed to explore the arena for 10 min. We used four parallel arenas, allowing four birds to be tested simultaneously. From the videos, number of dish visits, number of interactions with a dish, time spent in freeze response, and number of escape attempts was scored. A picture of the test set up (Fig. S11), as well as an ethogram with scoring criteria, is available in Supplementary Information 1.

### Exploration versus social test

Day 43–45, the chickens were tested in an L-shaped arena, which offered the options to either explore or to be social. In the right arm, the birds could see their conspecifics and interact with them through wire mesh, and in the left arm, containing two new novel objects and a dish with sand and 5 g of mealworms, the birds could explore and forage out of sight of the conspecifics. Four or five birds at a time were placed in the conspecific area and were allowed to habituate for 15 min. Thereafter, one individual at a time was taken out and placed in the start box. Each test session lasted 10 min and started when the sliding door was opened. The sessions were video recorded using a GoPro Hero 10 black camera attached for an overhead view. From the videos, latency to emerge, latency to enter social arm, latency to enter food arm, time spent in social arm, time spent in food arm, and number of zone crossings was scored. A picture of the test set up (Fig. S12), as well as an ethogram with scoring criteria, is available in Supplementary Information 1.

### Sampling and data analysis

For the video stimulation experiment, all three total play scores, i.e. video condition, pair condition and the mean play score, were used when assessing how play correlated with affective state. For the experiment investigating individual consistency in play, a play score was created for each individual and play category by taking the sum of the play frequency of the first and second day both weeks, i.e. the sum of four out of the six days. The play peak occurred the first week of testing and given the strong correlation between the first and second day for both sexes this week, it was decided to use these days from both weeks to create the play scores. The total play score was then used when assessing how play correlated with affective state and personality.

As a measurement of the affective state of the birds, we calculated a pessimism index from the cognitive judgement bias test in line with the method used previously by Lalot, et al.^38^:

$$\text{Pessimism index}=\frac{\text{Morph }-\text{ Mirror}}{\text{Owl }-\text{ Mirror}}$$

The latency time to approach the morph (ambiguous), the mirror (the most positive stimulus) and the owl (negative) were used. A high pessimism index reflects that the bird took longer time to approach the ambiguous stimulus, and presumably, were more pessimistic. Opposite, a low pessimism index reflects a shorter latency time to approach the ambiguous stimulus, indicating an optimistic state.

As for the three personality tests, the different variables were scored using Observer XT version 14. A principal component analysis (PCA; without rotation) including all the variables was performed. Three components were extracted and the factor scores of each component were saved. As for the relationship between play, affective state and personality, the correlation between the total play scores, the pessimism index and the factor scores, were assessed using Pearson’s correlation analysis. The graphs were made in R using the ggplot2 package, and the statistical analyses were performed in SPSS.

## Results

### Relationship between play and affective state

**Fig. 7:**
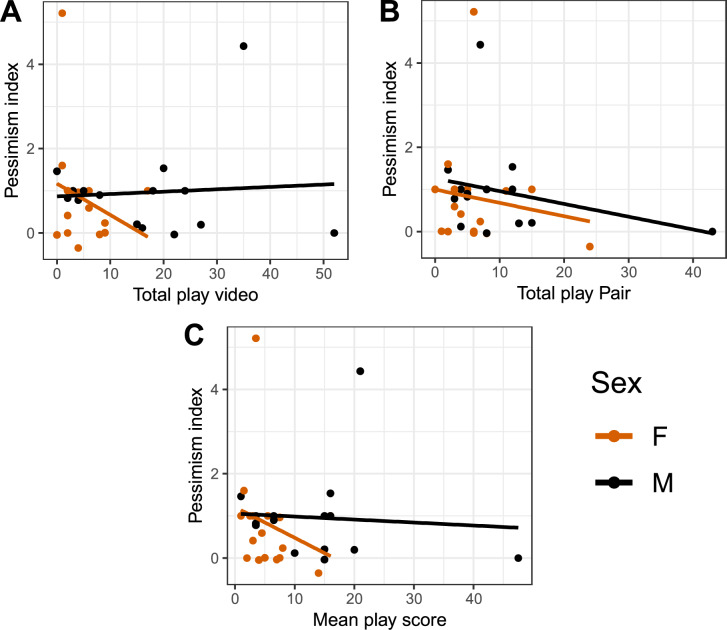
Correlations between total number of play observations per individual per 15 min and affective state, for the (**A**) video condition, (**B**) pair condition, and (**C**) mean play score of both conditions. Each dot represents one individual.

For the video stimulation experiment, no correlation between total play and affective state was found for either sex in the video condition (Fig. 7A; males: r = 0.07, p = 0.79; females: r = − 0.26, p = 0.32), the pair condition (Fig. 7B; males: r =  − –0.28, p = 0.32; females: − 0.15, p = 0.57), or with the mean play score (Fig. 7C; males: r =  − 0.08, p = 0.79; females: r =  − 0.24, p = 0.36).

**Fig. 8:**
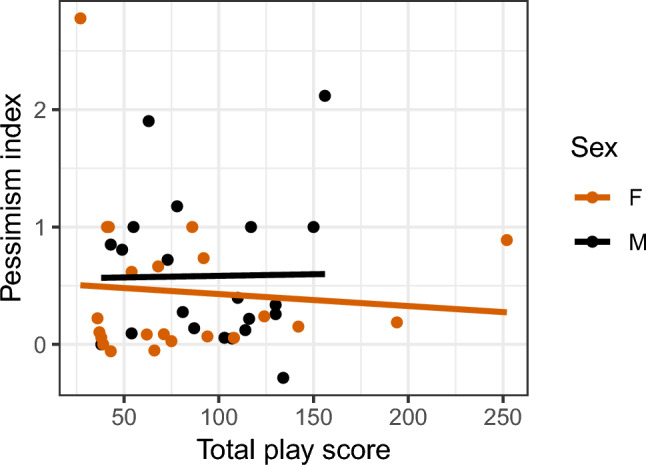
Correlation between affective state and total play. The play score is based on the sum of the play frequency of day 1 and day 2 from the two weeks of play tests. Each dot represents one individual.

For the individual consistency experiment, no correlation between either of the play categories and affective state was found (Fig. 8, total play; males: r = 0.02, p = 0.95; females: r = − 0.09, p = 0.69; Figure S9B, object play; males: r = 0.09, p = 0.7; females: − 0.03, p = 0.9; Figure S9C, locomotor play; males: r =− 0.26, p = 0.25; females: r =  − 0.27, p = 0.23; Figure S9D, social play; males: r = 0.13, p = 0.57; females: r = − 0.01, p = 0.95).

### Personality traits

The component matrix of the PCA is presented in Table 1. The scree plot revealed a markedly decrease in explained variance after three principal components, and three components were therefore extracted. Of the variance, the first component explained 27.9%, the second component 17.6%, and the third component 12.6%, resulting in a total of 58.1%. We labelled the first dimension “fearfulness”, the second “food exploration” and the third “sociability”, based on the variables with the highest loadings on each component.

**Table 1:** Principal component matrix of the variables scored in the three personality tests.

Test	Variable	Component 1	Component 2	Component 3
Social reinstatement	latency to leave start zone	0.455	0.420	− 0.614
	latency to enter social zone	0.436	0.480	− 0.672
	latency to first zone crossing	0.348	0.174	− 0.112
	number of zone crossings	− 0.387	− 0.098	0.344
	time spent in social zone	0.103	− 0.380	0.442
Exploration	number of dish visits	− 0.639	− 0.018	0.055
	number of interactions with a dish	− 0.681	0.239	0.192
	time in freeze response	0.510	− 0.092	0.169
	number of escape attempts	0.537	− 0.006	0.356
Exploration vs Social: L-arena	latency to emerge	0.769	0.381	0.384
	latency to enter social arm	0.492	0.591	0.466
	latency to enter food arm	0.699	− 0.544	− 0.020
	time spent in social arm	− 0.115	− 0.862	− 0.405
	time spent in food arm	− 0.635	0.584	0.039
	number of zone crossings	− 0.595	0.318	− 0.093

### Relationship between play and personality

**Fig. 9:**
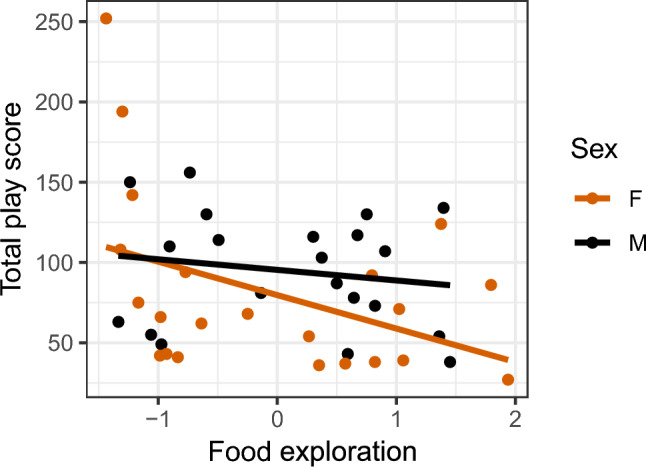
Correlation between and total play and food exploration. The total play scores are based on the sum of the play frequency of day 1 and day 2 from the two weeks of play tests. Each dot represents one individual.

For females, there was a significant negative correlation between total play and food exploration (Fig. 9, males: r = − 0.17, p = 0.47; females: r = − 0.42, p = 0.05). No correlation was found between either total play and fearfulness (males: r = − 0.29, p = 0.2; females: r = 0.09, p = 0.7), or total play and sociability (males: r = 0.06, p = 0.81; females: r = − 0.09, p = 0.67). How the different play categories correlates to the personality traits is shown in figure S13, Supplementary Information 1.

### Relationship between affective state and personality

**Fig. 10:**
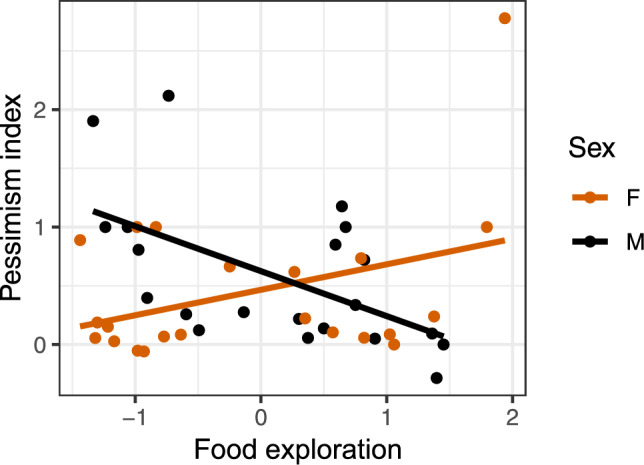
Correlation between affective state and food exploration. Each dot represents one individual.

For males, there was a significant negative correlation between affective state and food exploration (Fig. 10, males: r = -0.56 p = 0.01; females: r = 0.37, p = 0.09). No correlation was found between affective state and fearfulness (males: r = 0.12, p = 0.6; females: r = -0.21, p = 0.35), or affective state and sociability (males: r = -0.38, p = 0.09; females: r = 0.01, p = 0.98).

## Discussion

In Part 3, we aimed to assess the relationship between play, affective state and personality. Regardless of play stimulation method and play category, no consistent relationship between play frequency and affective state was found for either males or females. Three main personality traits emerged from the analysis, namely fearfulness, food exploration and sociability. Food exploration was found to be negatively correlated with total play in females and negatively correlated with affective state in males, indicating that low playing females were more food motivated than high playing females, and optimistic males were more food motivated than pessimistic males.

Our results contradict other studies, where connections between positive affective state and object play^18^, locomotor play^17^, and social play^39^ have been found. In these studies, different behavioural indicators, a combination of behavioural and physiological measures, or administration of morphine, was used to either identify or induce a more positive affect. In neither of these studies were judgement bias used as a measure of the affective state of the animals, which could be one reason for the different results found here. Collecting multiple measures indicative of affective state could strengthen results, since without knowledge of how different measures correlates within species, comparing between studies and across species becomes more difficult.

It is also possible that the outcome of the cognitive judgement bias test may not reflect an underlying personality trait, but rather a context-based response. In humans, cognitive judgement bias is considered to have elements of both an underlying trait and a transient state^40^. A trait reflects a consistent individual tendency toward pessimism or optimism, while a state refers to the fluctuating levels of pessimism or optimism that can vary depending on the situation or context. Applied to non-human animals, the way cognitive judgement bias is often tested, it most likely reflects a situation-specific measure of expectation, i.e., a state rather than a trait. However, recent studies have found optimism levels to be stable over time in rats^41^ and dairy calves^42^, and in chickens, early stress from commercial hatchery processing induces increased short and long-lasting pessimism^26^. Contradicting this, Rygula, et al.^41^ and Quante, et al.^43^ did not find optimism levels to be stable over time in rats. Hence, there is a need for further research into potential factors that could influence or alter the stability of optimism levels both within and across various stages of life.

Low playing females showed higher levels of food exploration than high playing females. In general, some individual variation in motivation to explore for food is expected to occur, and it is likely that high food motivation suppresses the motivation to play, as play is known to mainly occur when basic needs are met. However, the birds were not deprived of food before any of the tests. For the play tests, all individuals were assumed to have had about the same degree of satiation, but in for instance the Social versus Exploration test, four or five individuals were tested at a time, and whether they were tested first or last could have influenced their motivation to forage.

Optimistic males were found to be more food explorative than pessimistic males. This is in line with expectations, as optimistic individuals supposedly have a higher anticipation for positive outcomes, hence have a higher expectation for presence of food. The absence of this pattern in females is more difficult to interpret. Apart from the finding in males, no other connection between affective state and personality was found. This could indicate that there are weak or no connections between affective state and the observed personality traits in chickens, or the outcome of the tests do not actually reflect the traits we attempted to quantify. Similar to our results, Lalot, et al.^38^ found no connection between cognitive bias and different personality traits in canaries. In mammals, findings regarding the affective state-personality relationship varies. Personality has been found to play a key part in the cognitive processing in domestic dogs^44^. For example, sociability and excitability were associated with an optimistic bias, and anxiety and fear with a pessimistic bias. In lambs, a reduction of fearfulness by administration of diazepam, a benzodiazepine normally used to reduce negative affective state, seemed to induce a more optimistic state^45^. Whether or not connections are present in some species but not in others, or if differences in methodology are influencing the results remains to be explored.

Our results suggest that fundamental affective state is weakly associated with play motivation in the present setup. However, our method for assessing affective state may not have been sensitive enough. There is a need for better understanding of how different measures of affective state correlate, and whether they indicate a short or long-term reflection of the affective state of an animal. Other ways of measuring affective state in chickens should be explored, compared and related to play, to explore the relationship further.

## General discussion and conclusions

In separate experiments, we have evaluated two different methods as potential ways of studying individual variation in play in young chickens; video stimulation and individual consistency in play, as well as explored the link between individual motivation to play, affective state and personality. Video stimulation showed potential to stimulate play, but the play frequency of the chicks was quite low overall, and the method elicited no social play. Regardless of group constellation, the chicks played more when individual consistency in play was investigated compared to when video stimulation was used, presumably because we were unsuccessful in replacing the comfort of being in a group with the video. Furthermore, this method allowed for behaviours of all play categories to be captured. The individual play frequency was found to be relatively consistent over the three test days, suggesting that there is a genetic basis for variation in play motivation. Although context specific, we believe that this method gives a reliable measure of the individual motivation to play, and that two days of testing is enough to capture short-term consistency.

The finding that individual playfulness is somewhat consistent has important implications for animal welfare. As pointed out by Lampe, et al.^15^, provided that engaging in play is linked to the experience of positive emotions, then a measure of an individual’s motivation to play could predict the welfare of that individual over some time and not only in a particular moment. However, our results do not show that individual play motivation is associated with a fundamental state of affect, but this requires further research. Lambs show positive anticipation for opportunities to play^46^, indicating that play is associated with a positive welfare state. Hence, regardless of if the fundamental state of animals is rather negative or positive, individuals are likely to have positive experiences during the execution of play behaviour. If animals play a lot in their home pens, this is then likely to reflect a more positive welfare state in that environment.

In previous studies on chickens, broiler chicks kept in a barren environment were found to play more when given the possibilities than those in an enriched environment^47^, and we previously reported that laying hen chicks exposed to early stress play more than non-stressed chicks^4^. These findings suggest that a greater contrast between home pen environment and play situation stimulates more play, and similarly, that chicks in a more negative state of mood experience a more intense stimulation when offered play opportunities. Hence, the opportunity to play is supposedly enriching for the birds and has greater effect on animals with compromised welfare. In the current study, the chicks were moved out of their home environment during play sessions. Consequently, birds in a more negative affective state may have played more in the play arenas compared to birds in a more positive affective state. For future studies, investigating how the individual play frequency in the home pen correlates with measures of affective state could add valuable knowledge about the play-affective state relationship.

## Supplementary Information

Supplementary Information 1.

Supplementary Information 2.

Supplementary Information 3.

Supplementary Information 4.

## Data Availability

The complete datasets generated during the included studies are provided in Supplementary Information 2.
