# Supplementary material for: Individual variation in play in young chickens – assessment and connection to affective state and personality
Source: Sci Rep. 2026 Jan 6;16:634. doi: 10.1038/s41598-025-34437-x (PMC12775386; doi:10.1038/s41598-025-34437-x)
Supplement: Supplementary file 1 — Supplementary Information 1. [file 41598_2025_34437_MOESM1_ESM.pdf]

Individual variation in play in young chickens – assessment and connection to affective state and personality

Oscarsson, Rebecca; Hedlund, Louise; Rutkauskaite, Austeja; Jensen, Per

Supplementary material Part 1

Supplementary information:

Figure S1: Video stimulation test set-up from above.

Figure S2: Video stimulation set-up.

Figure S3: Correlations between the video and pair condition for the different play categories.

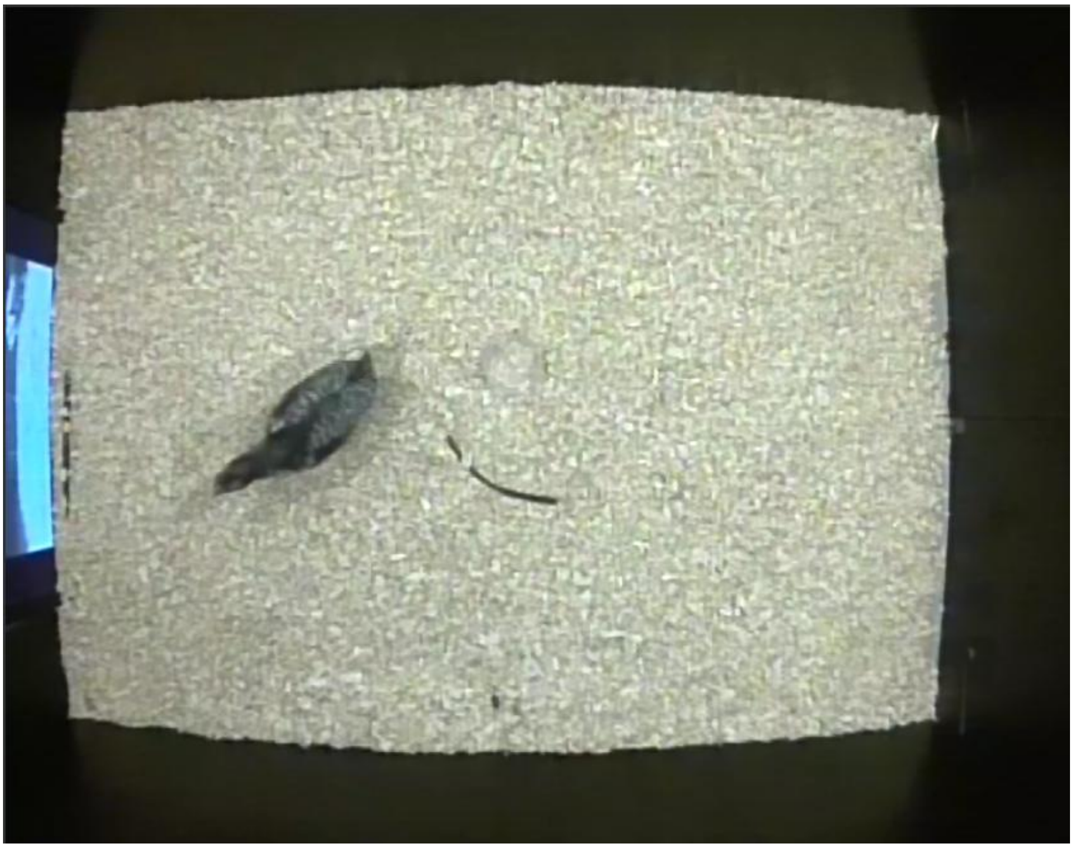

Figure S1. Picture of the video stimulation test set-up viewed from above. Taken during a test (day 28). In the middle of the arena is a rubber worm and a transparent bowl with mealworms. To the left is the screen which shows the play stimulation video.

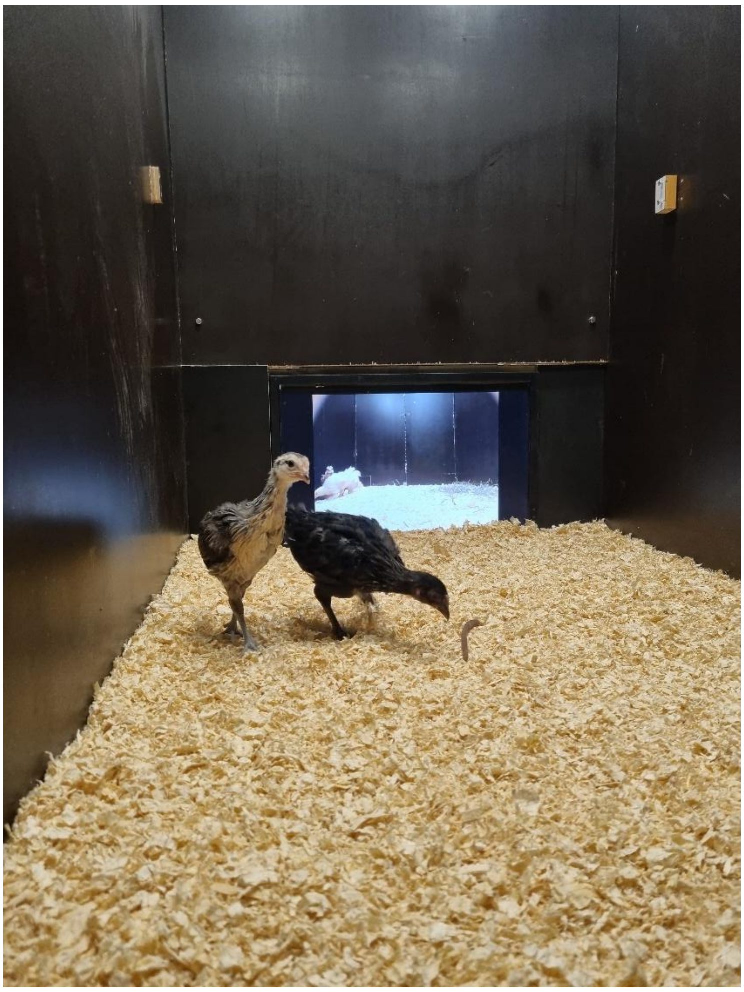

Figure S2. Picture of the video stimulation set-up. Note that the photo is taken post end of experiment.

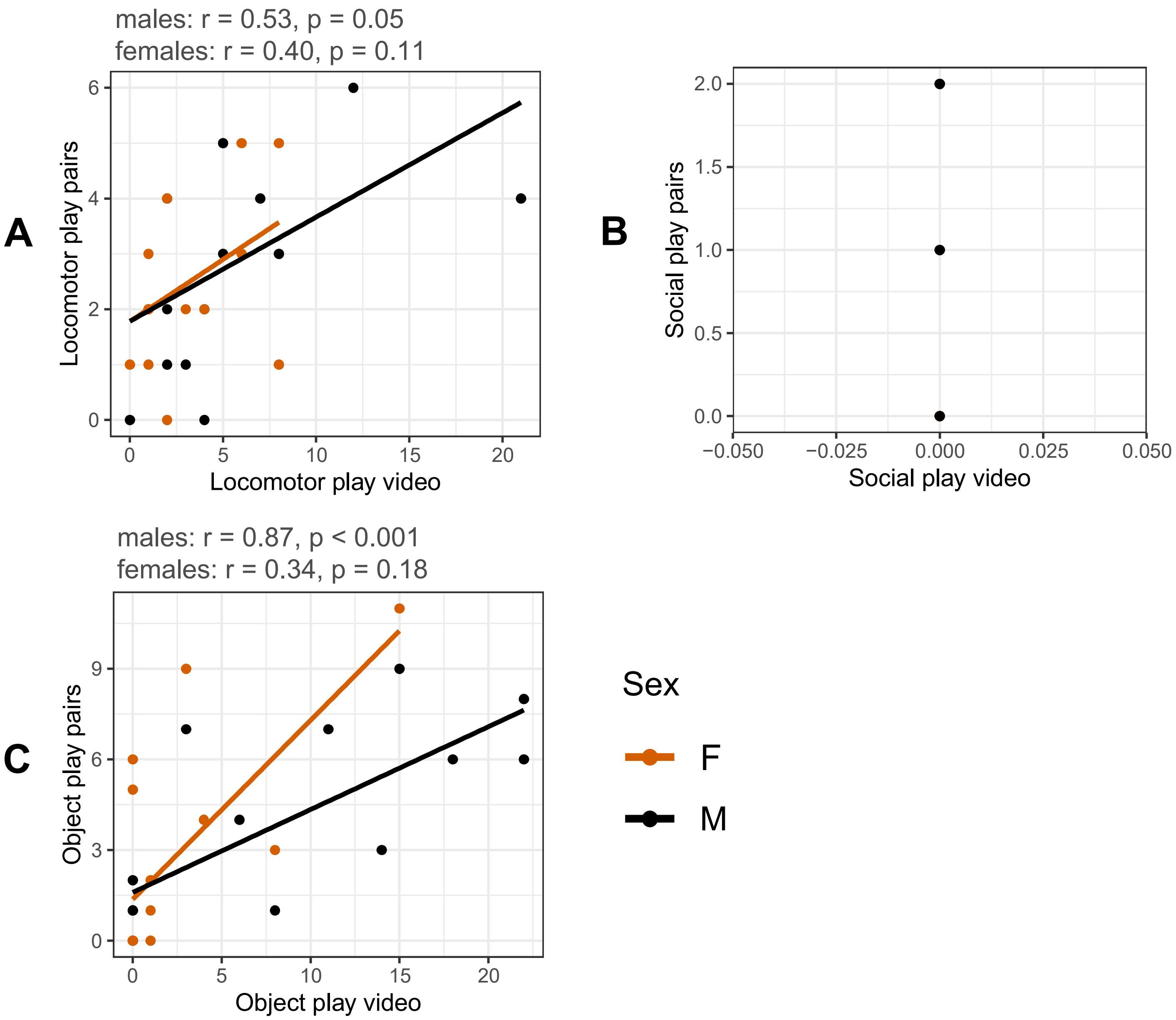

Figure S3. Correlations between total number of **(A)** locomotor, **(B)** social, and **(C)** object play observations in the video and pair condition per 15 min. Each dot represents one individual. No statistical analysis could be performed for social play as it was only performed by two males in the pair condition.

Supplementary material Part 2

Supplementary information:

Figure S4: Correlations for object play between the three test days.

Figure S5: Correlations for locomotor play between the three test days.

Figure S6: Correlations for social play between the three test days.

Figure S7: Correlations between different play types.

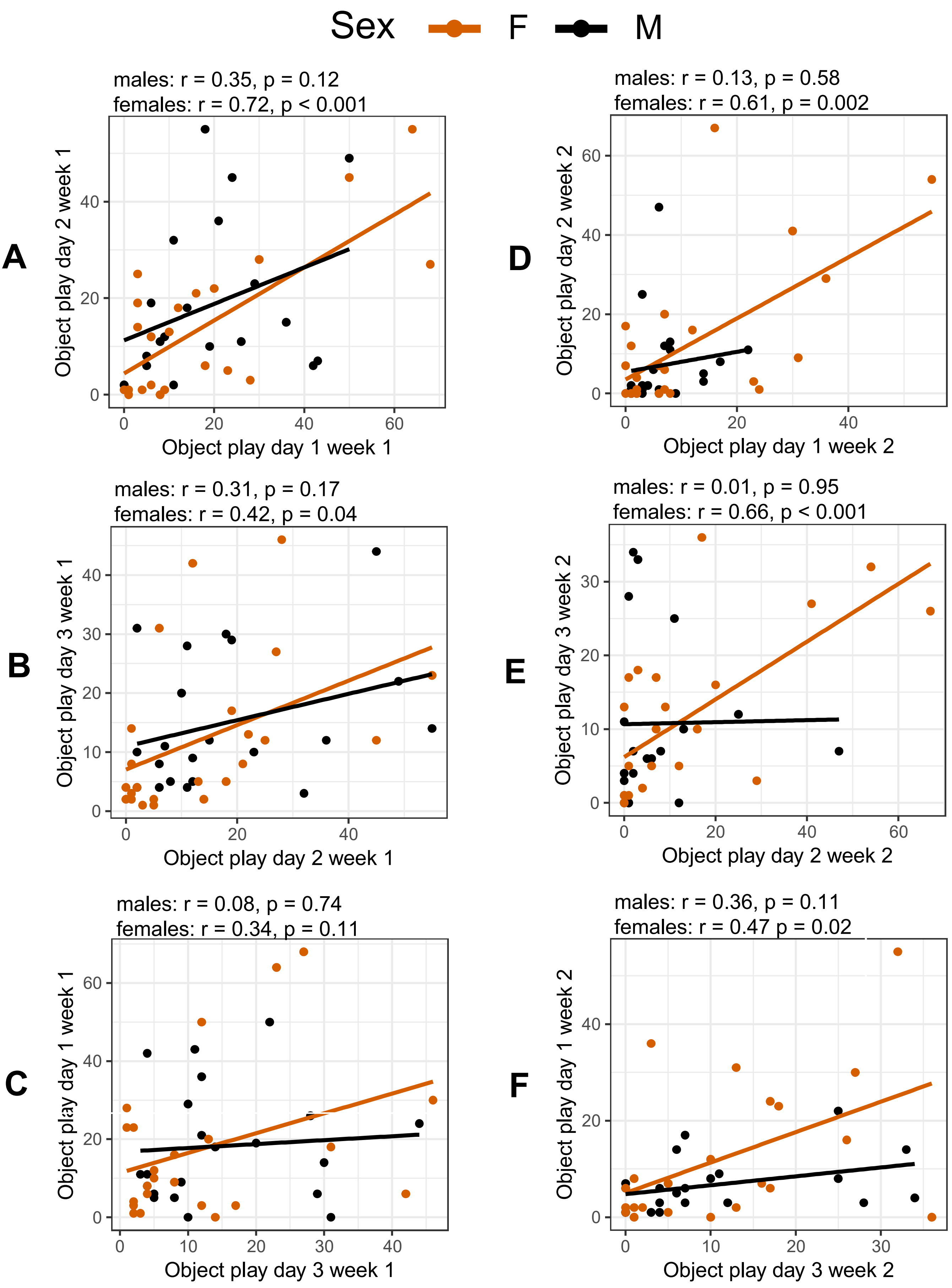

Figure S4. Correlations between total number of object play observations per individual per 15 min of **(A)** day 1 and 2 week 1, **(B)** day 2 and 3 week 1, **(C)** day 3 and 1 week 1, **(D)** day 1 and 2 week 2, **(E)** day 2 and 3 week 2, **(F)** day 3 and 1 week 2.

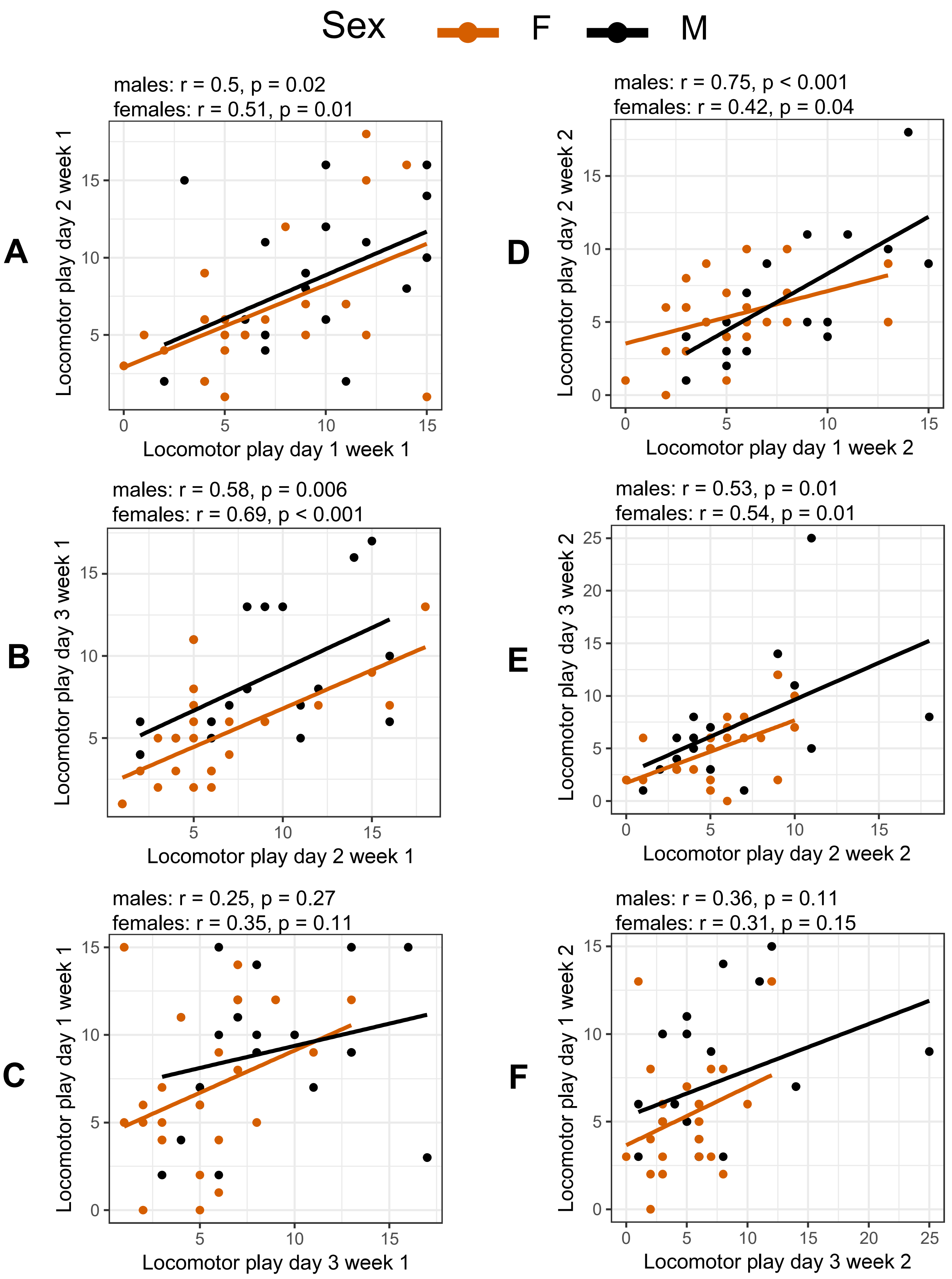

Figure S5. Correlations between total number of locomotory play observations per individual per 15 min of **(A)** day 1 and 2 week 1, **(B)** day 2 and 3 week 1, **(C)** day 3 and 1 week 1, **(D)** day 1 and 2 week 2, **(E)** day 2 and 3 week 2,**(F)** day 3 and 1 week 2.

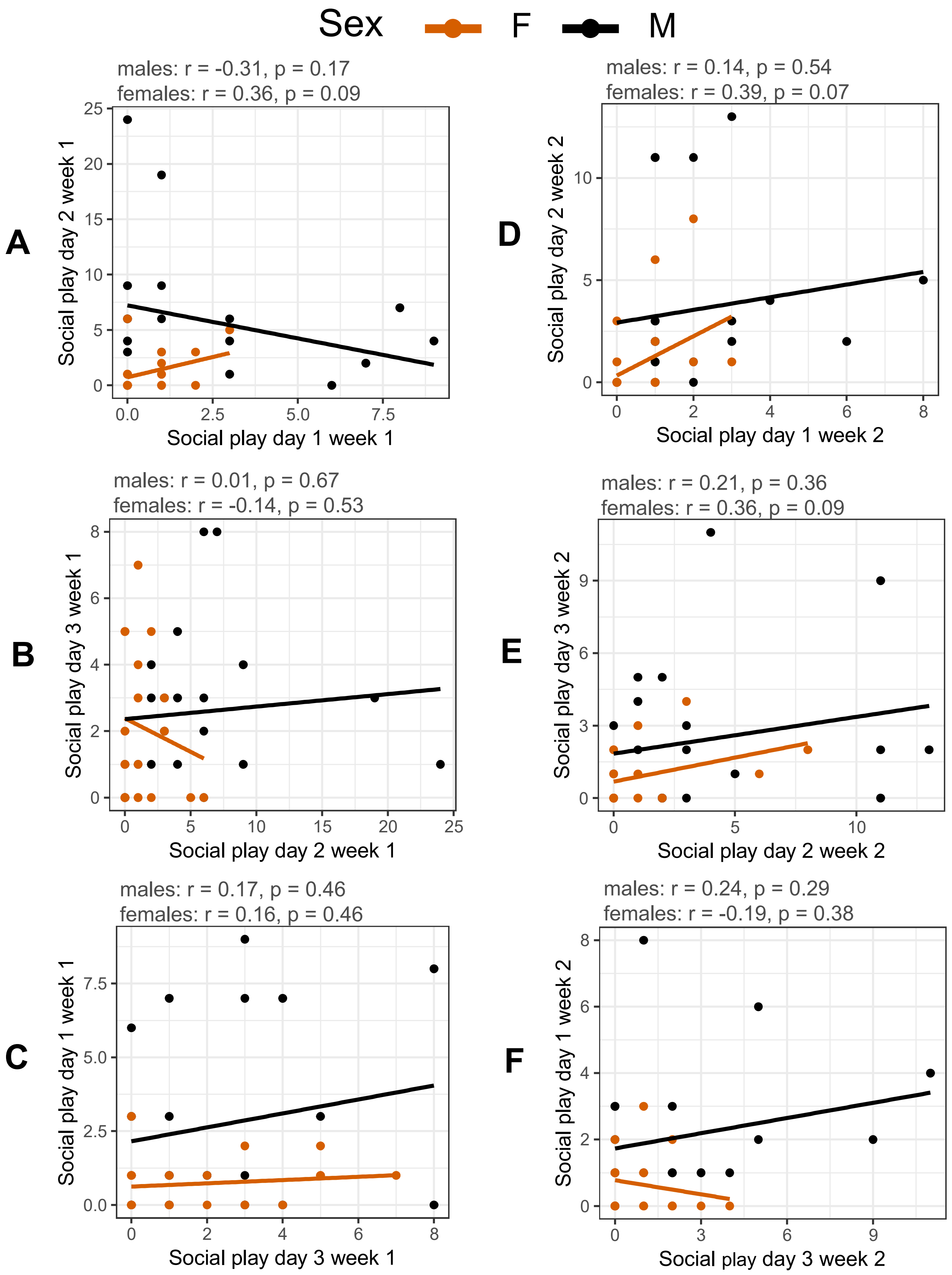

Figure S6. Correlations between total number of social play observations per individual per 15 min of **(A)** day 1 and 2 week 1, **(B)** day 2 and 3 week 1, **(C)** day 3 and 1 week 1, **(D)** day 1 and 2 week 2, **(E)** day 2 and 3 week 2, **(F)** day 3 and 1 week 2.

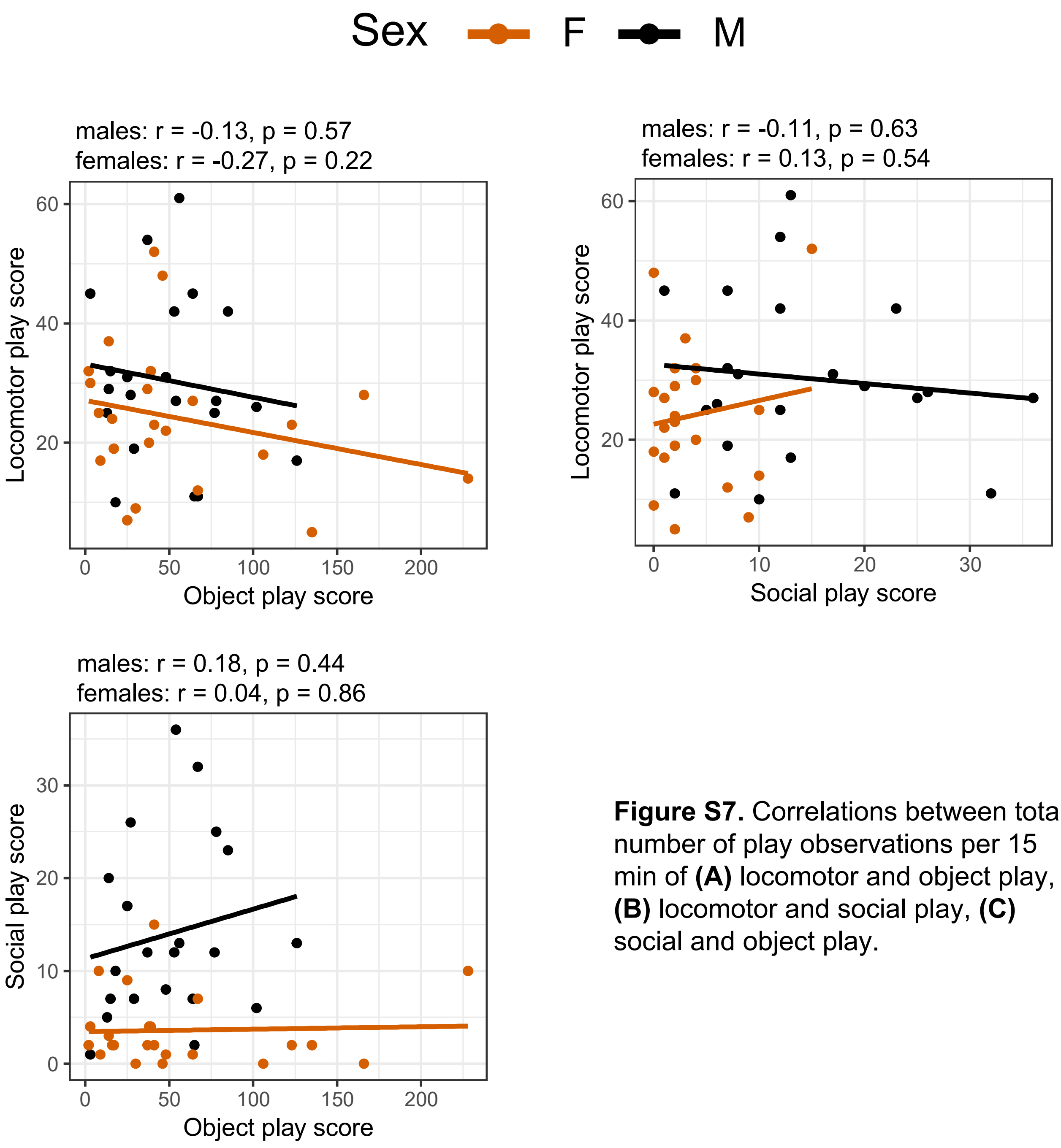

Figure S7. Correlations between total number of play observations per 15 min of **(A)** locomotor and object play, **(B)** locomotor and social play, **(C)** social and object play.

Supplementary material Part 3

Supplementary information:

Figure S8: Cognitive judgement bias stimuli

Figure S9: Correlations between the different play categories and affective state

Figure S10: Social reinstatement set-up

Figure S11: Exploration test set-up

Figure S12: Exploration vs Social test set-up

Figure S13: Correlations between personality traits (factor scores) and the different play categories.

Cognitive judgement bias test

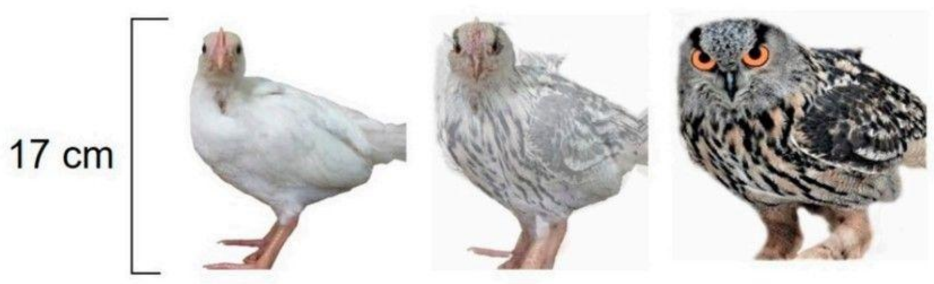

Figure S8. The three stimuli used for cognitive judgement bias test: chick (near positive), morph (ambiguous) and owl (negative). The figure is from Hedlund et al. (2022), as the same stimuli were used.

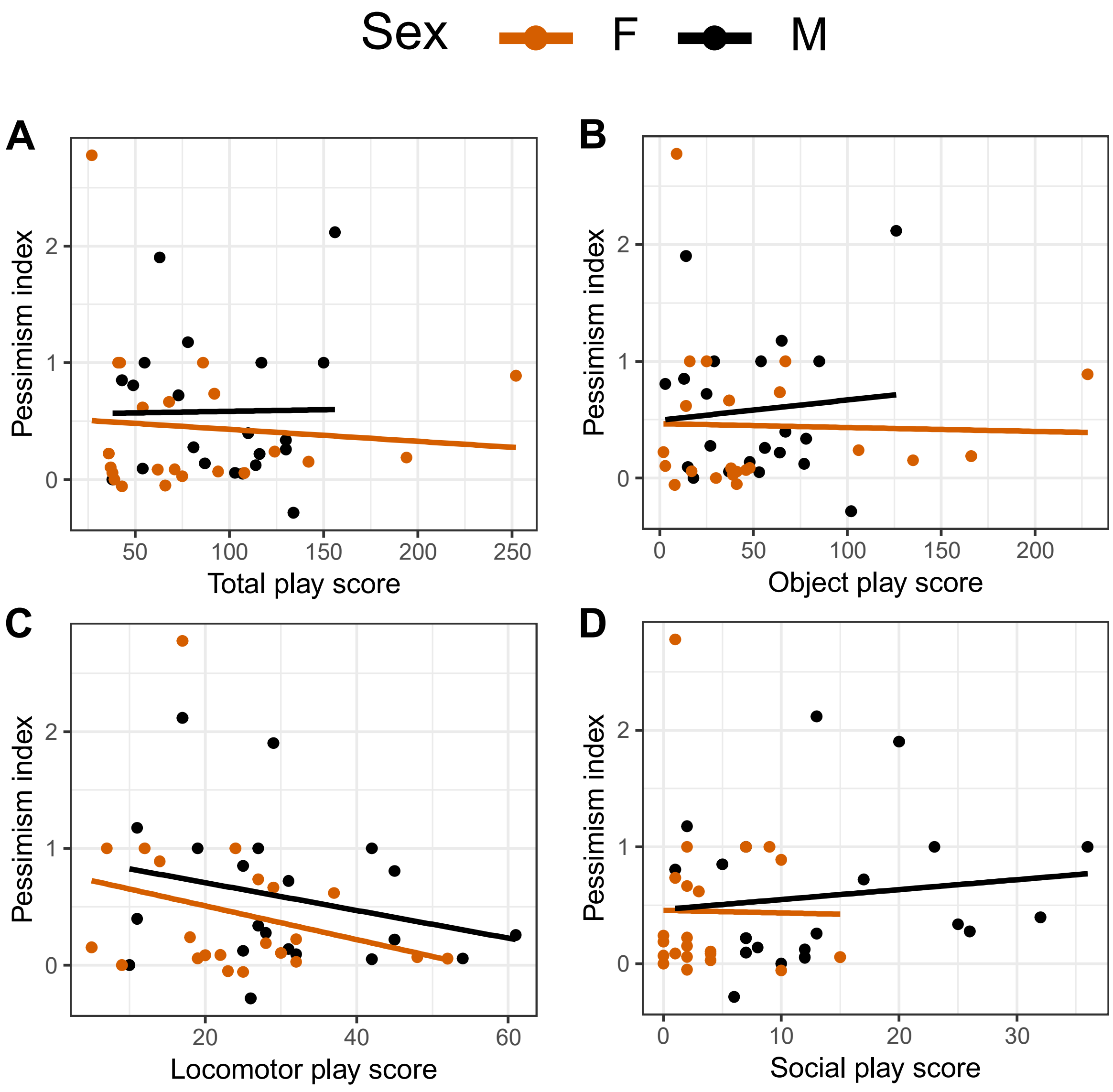

Figure S9. Correlations between total number of play observations per individual per 15 min and affective state, for **(A)** Total play, **(B)** object play, and **(C)** locomotor play, and **(D)** Social play. The play scores are based on the sum of the play frequency of day 1 and day 2 from the two weeks of play tests. Each dot represents one individual.

Personality tests - Ethograms and scoring criteria

Test 1: Social reinstatement

Observation time: 600s.

Observation start is when the light is turned on.

Behaviour / Variable name	Sampling	Description and scoring criteria
Latency to leave start zone	Time-point	Time point at which the bird leaves the start zone. Scored only once the head and base of neck/shoulder are outside of the start zone.
Latency to enter social zone	Time-point	Time point of entering the social zone for the first time. Scored only once the head and base of neck/shoulder are within the social zone.
Latency to first zone crossing	Time-point	Time point at which the bird leaves the social zone for the first time. Scored once the head and base of neck/shoulder are outside of the social zone.
Time spent in social zone	Continuous	Amount of time in seconds the bird is located inside the social zone. See behaviour above for scoring criteria.
Zone crossings	Total #	Number of times the bird had crossed the imaginary line in or out of the social zone. Crossings are scored only once the head and base of neck/shoulder are past the line.

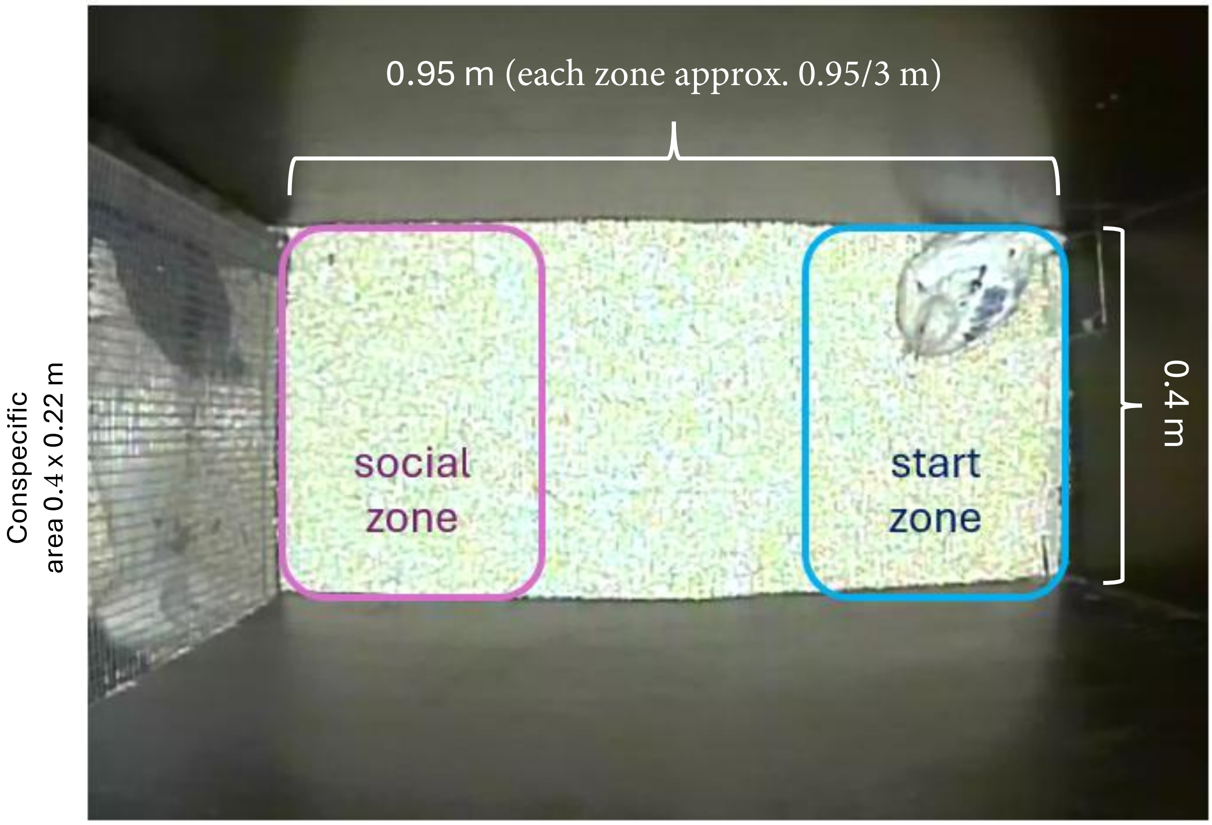

Figure S10. Picture of the Social reinstatement set-up with the different zones used when scoring marked. Note that the zones were not visible to the birds.

Test 2: Exploration arena

Observation time: 600s.

Observation start is at the point of lights turning on. Dishes A and C were baited with mealworms.

Behaviour / Variable name	Sampling	Description and scoring criteria
Dish visit	Total #	Number of times the bird had visited a dish. A dish visit is scored as the head and/or neck overlapping the diameter of the dish. Not counted if the bird simply runs over the dish without orientating its head/neck towards it.
Interaction with a dish	Total #	Number of times the bird, during a visit, physically interacted with the dish or its contents (pecking, scratching).
Freeze response	Continuous	Time spent in freeze response. The bird was considered to be in freeze when completely still for more than five seconds.
Escape attempts	Total #	Number of times the bird jumped up toward the walls of the arena, seemingly looking for a way out.

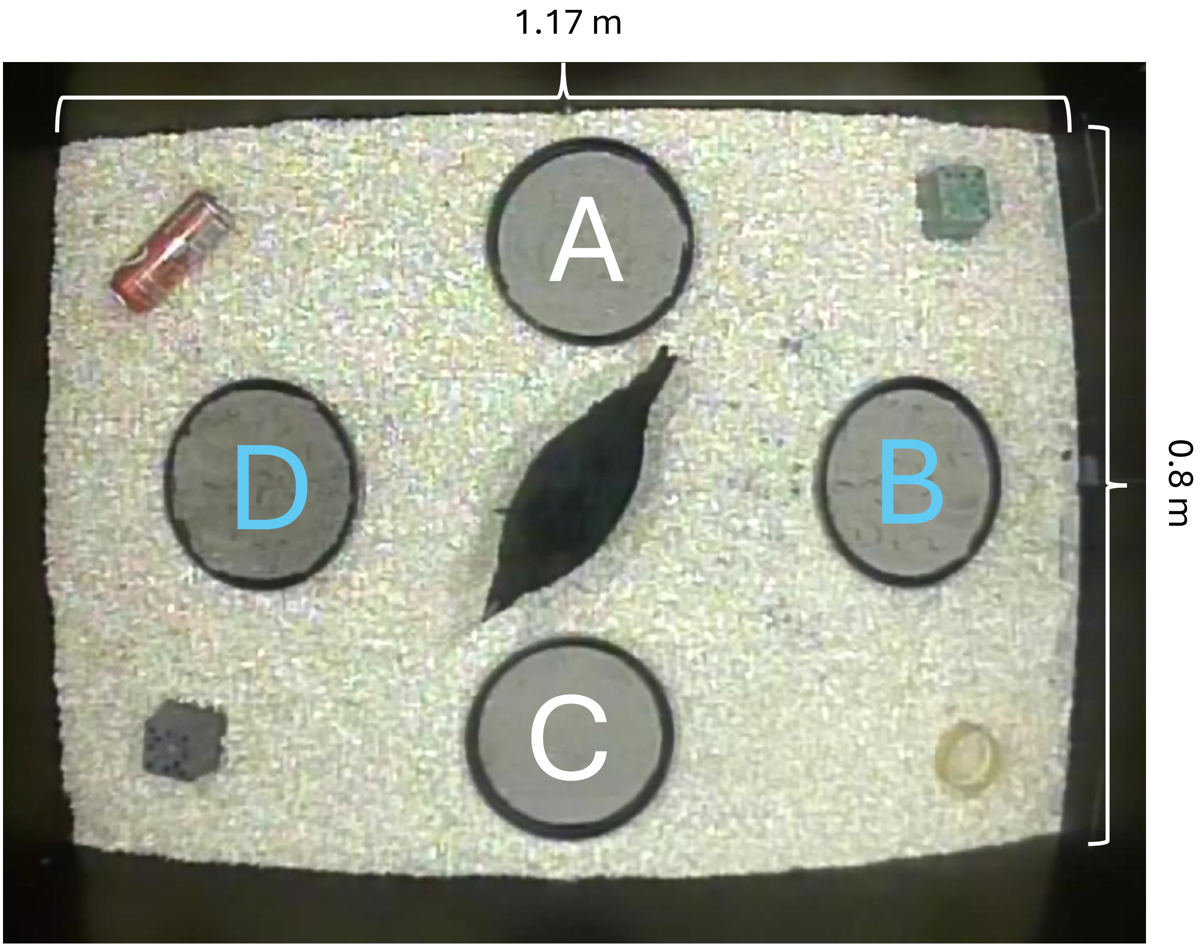

Figure S11. Picture of the Exploration test set-up. Dish A-D were filled with sand, and in addition, dish B and D were bated with mealworms. One novel object was placed in each corner.

Test 3: Social vs Exploration test (L-shape arena)

Observation time: 600s.

Observation start is at the opening of the start box.

Behaviour / Variable name	Sampling	Description and scoring criteria
Latency to emerge	Time-point	Time point at which the bird leaves the start box. Scored only once the head and base of neck/shoulder are outside of the start box.
Latency to enter social arm	Time-point	Time point of entering the social arm for the first time. Scored only once the head and base of neck/shoulder are within the social arm.
Latency to enter food arm	Time-point	Time point of entering the food arm for the first time. Scored only once the head and base of neck/shoulder are within the food arm.
Time spent in social arm	Continuous	Amount of time in seconds the bird is located inside the social and semi-social zone combined. Scored only once the head and base of neck/shoulder are within the semi-social zone
Time spent in food arm	Continuous	Amount of time in seconds the bird is located inside the food and novel object zone combined. Scored only once the head and base of neck/shoulder are within the novel object zone.
Zone crossings	Total #	Number of times the bird had crossed the imaginary line in or out of any of the 5 zones. Crossings are scored only once the head and base of neck/shoulder are past a line.

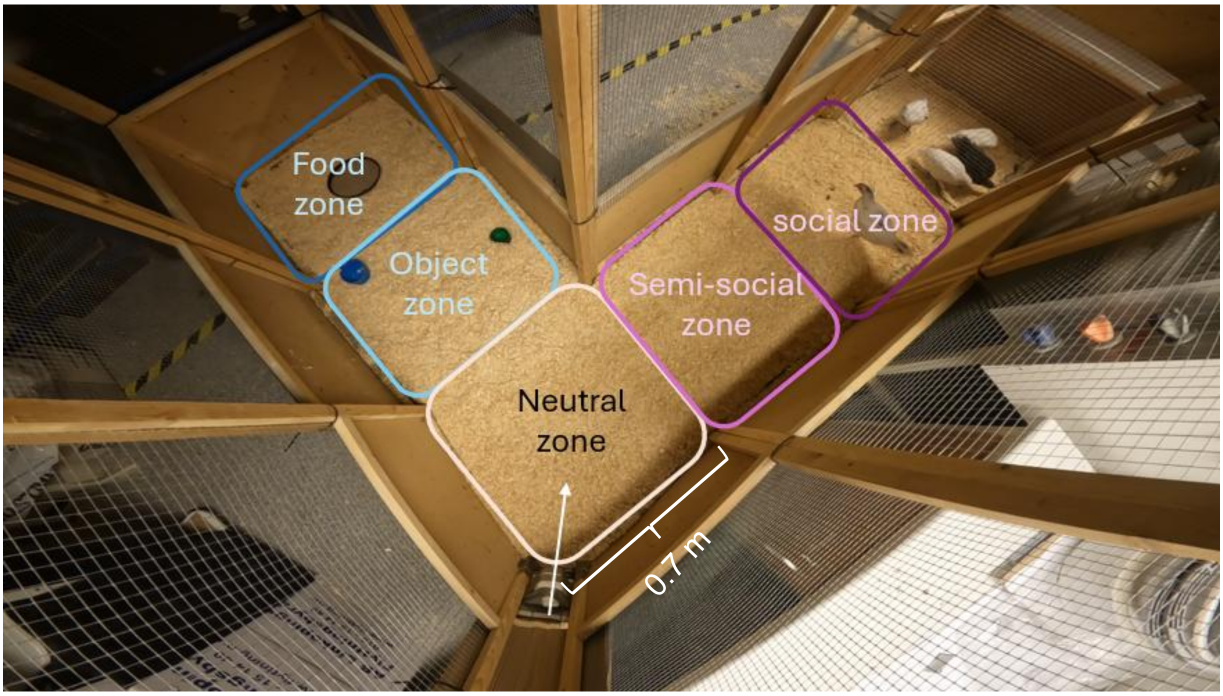

Figure S12. Picture of the Exploration vs Social test set-up. The object zone contained two novel objects (different from the ones used in the Exploration test). The dish in the food zone contained sand and was bated with 5 g of mealworms. All zones are square and have the same measurements.

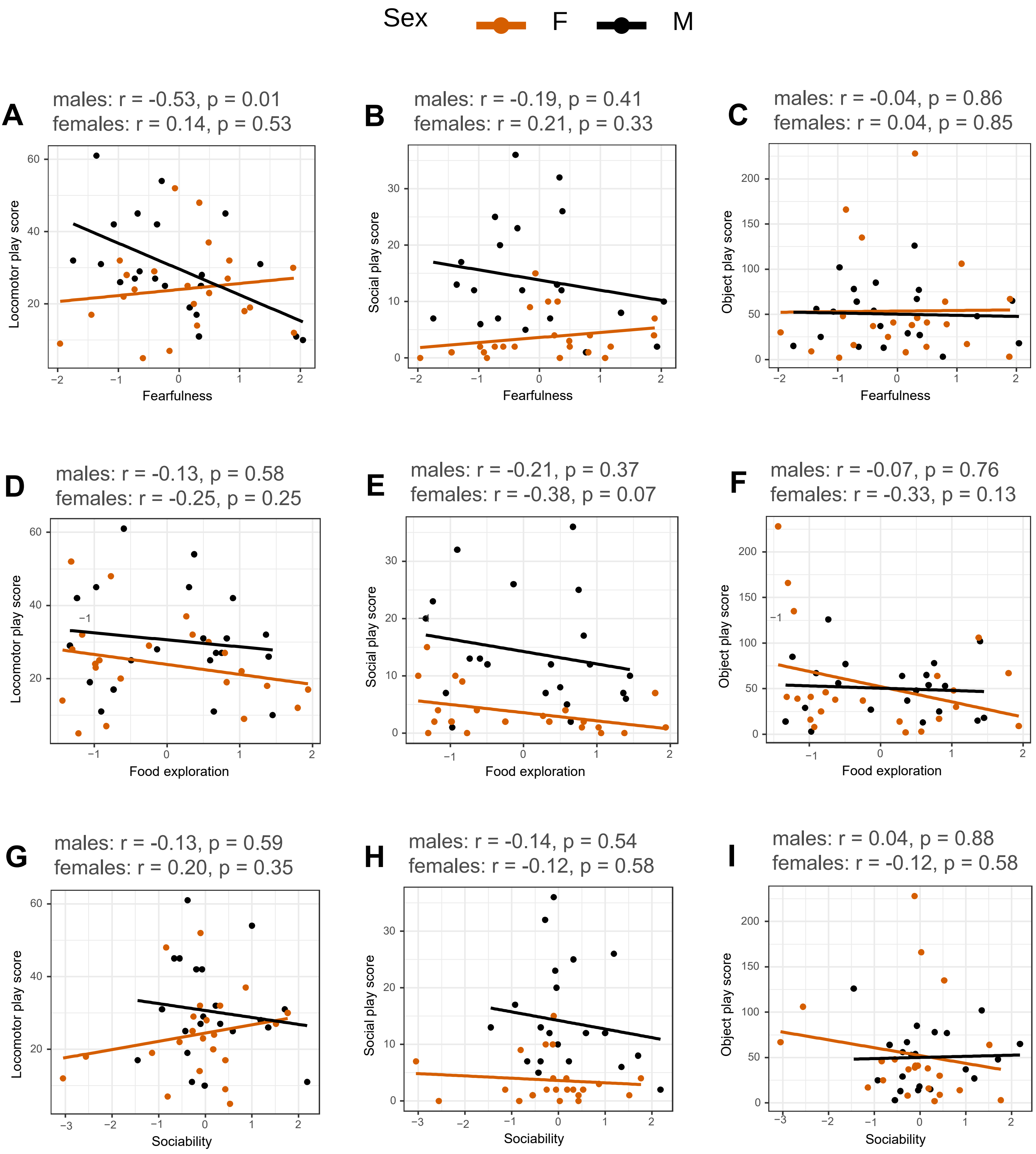

Figure S13. Correlations between fearfulness and number of observations per individual per 15 min of **(A)** locomotor, **(B)** social**, (C)** object play. Correlations between food exploration and number of observations per individual per 15 min of **(D)** locomotor, **(E)** social, **(F)** object play. Correlations between sociabilityand number of observations per individual per 15 min of **(G)** locomotor, **(H)** social, **(I)** object play.
