# Supplementary material for: Individual variation in play in young chickens – assessment and connection to affective state and personality
Source: Sci Rep. 2026 Jan 6;16:634. doi: 10.1038/s41598-025-34437-x (PMC12775386; doi:10.1038/s41598-025-34437-x)
Supplement: Supplementary file 3 — Supplementary Information 3. [file 41598_2025_34437_MOESM3_ESM.pdf]

Individual variation in play in young chickens – assessment and connection to affective state and personality

Oscarsson, Rebecca; Hedlund, Louise; Rutkauskaite, Austeja; Jensen, Per

Supplementary information:

Table S1: The complete play ethogram

Table S1. Ethogram for all observed play behaviours inside the test arenas divided into the corresponding categories of locomotor, social and object play. Definitions have been adapted from references [1-4].

**Locomotor play**	
Running	Spontaneous forward movement with at least twice the normal walking pace, often including rapid direction changes. Can start either while walking or being stationary. Wing flapping does not occur simultaneously.
Frolicking	Spontaneous and rapid running and/or jumping while wings either flapping or raised. Often with rapid direction changes. A frolicking bout ends when the bird resumes another activity.
Wing flapping	Rapid vertical movement with both wings while stationary or walking up to 2 steps. Excludes wing flaps performed by a bird to balance itself or correct its feathers.
Spinning	Circling movement around the birds own axis with at least twice the normal walking pace. Wing flapping does not occur simultaneously.

Spinning while wing flapping	Circling movement around the birds own axis with at least twice the normal walking pace. Wing flapping occurs simultaneously
**Social play**	
Sparring jumping, with no contact	Simulation of fighting behaviour with no obvious aggression. Two birds jumping while standing close facing one another. Jumping may also be performed by one bird while the other bird is passive or displays light avoidance. Wings flapping, extended, or kept to the sides. May include light kicking. No physical contact involved.
Sparring jumping, with contact	Simulation of fighting behaviour with no obvious aggression. Two birds jumping while standing close facing one another. Jumping may also be performed by one bird while the other bird is passive or displays light avoidance. Wings flapping, extended, or kept to the sides. May include light kicking. Involves physical contact.
Sparring stand-off, with no contact	Simulation of fighting behaviour with no obvious aggression. The following behaviours may occur during a bout: birds face each other briefly, stepping close and backing off from one another. While stepping close, necks and feathers around the neck are often raised. Pecking towards neck, head, or beak of the receiving bird. Wings are either flapping, extended or kept to the sides. Involves no physical contact.
Sparring stand-off, with contact	Simulation of fighting behaviour with no obvious aggression. The following behaviours may occur

	during a bout: birds face each other briefly, stepping close and backing off from one another. While stepping close, necks and feathers around the neck are often raised. Pecking towards neck, head, or beak of the receiving bird. Wings are either flapping, extended or kept to the sides. Involves physical contact.
**Object play**	
Object running	A bird picks up an object (normally the fake worm) in its beak and starts either walking or running. Rapid changes of pace or direction might occur. Other birds may begin to chase the bird carrying the object.
Worm running	A bird picks up a mealworm in its beak and starts either walking or running. Rapid changes of pace or direction might occur. Other birds may begin to chase the bird carrying the mealworm.
Object/worm chasing	A bird chases after an individual performing object- or worm-running and may try to obtain the carried object or worm.
Object/worm exchange	An object or a worm is obtained by one bird from another bird's beak. The object can be obtained either by a chasing bird from a running bird, or from a stationary bird with an object in its beak
Worm pecking	Bird pecks at fake worm or mealworm on the ground. The worm may be lifted off the ground and/or shaken but not carried.

References:

1 Baxter, M., Bailie, C. L. & O' Connell, N. E. Play behaviour, fear responses and activity levels in commercial broiler chickens provided with preferred environmental enrichments. *animal* **13**, 171-179, doi:10.1017/S1751731118001118 (2019).

2 Cloutier, S., Newberry, R. C. & Honda, K. Comparison of social ranks based on worm-running and aggressive behaviour in young domestic fowl. *Behav Processes* **65**, 79-86, doi:10.1016/j.beproc.2003.07.001 (2004).

3 Dawson, J. S. & Siegel P, B. Behavior Patterns of Chickens to Ten Weeks of Age. *Poultry Science* **46**, 615-622, doi:10.3382/ps.0460615 (1967).

4 Liu, Z., Torrey, S., Newberry, R. C. & Widowski, T. Play behaviour reduced by environmental enrichment in fast-growing broiler chickens. *Applied Animal Behaviour Science* **232**, doi:10.1016/j.applanim.2020.105098 (2020).
